# *Helicobacter pylori*, HIV and Gastric Hypochlorhydria in the Malawian Population

**DOI:** 10.1371/journal.pone.0132043

**Published:** 2015-08-05

**Authors:** Joe Geraghty, Alexander Thumbs, Anstead Kankwatira, Tim Andrews, Andrew Moore, Rose Malamba, Neema Mtunthama, Kai Hellberg, Lughano Kalongolera, Paul O’Toole, Andrea Varro, D. Mark Pritchard, Melita Gordon

**Affiliations:** 1 Malawi-Liverpool-Wellcome Trust Clinical Research Programme, PO Box 30096, Blantyre 3, Malawi; 2 Department of Clinical Infection, Microbiology and Immunology, Institute of Infection and Global Health, University of Liverpool, Liverpool, United Kingdom; 3 Department of Gastroenterology, Institute of Translational Medicine, University of Liverpool, Liverpool, United Kingdom; 4 Department of Gastroenterology, Royal Liverpool University Hospital, Liverpool, United Kingdom; 5 Department of Surgery, College of Medicine, Blantyre, Malawi; 6 Department of Medicine, College of Medicine, Blantyre, Malawi; 7 Department of Pathology, Royal Liverpool University Hospital, Liverpool, United Kingdom; 8 Department of Cellular and Molecular Physiology, University of Liverpool, United Kingdom; Liverpool School of Tropical Medicine, UNITED KINGDOM

## Abstract

**Background:**

HIV and *Helicobacter pylori* are common chronic infections in sub-Saharan Africa. Both conditions can predispose to gastric hypochlorhydria that may be a risk factor for enteric infections and reduced drug absorption. We have investigated to what extent HIV and *H*. *pylori* infections are associated with hypochlorhydria in a Malawian cohort of patients undergoing endoscopy.

**Methods:**

104 sequential symptomatic adults referred for gastroscopy at Queen Elizabeth Central Hospital, Blantyre, Malawi, had blood taken for rapid HIV testing and fasting serum gastrin analysis. Gastric fluid was aspirated for pH testing, and gastric biopsies were taken.

**Results:**

After 9/104 HIV-infected patients who were already established on anti-retroviral therapy were excluded, 17/95 (25.0%) were seropositive for untreated HIV, and 68/95 (71.6%) patients were *H*. *pylori* positive by histology. Hypochlorhydria (fasting gastric pH>4.0) was present in 55.8% (53/95) of patients. *H*. *pylori* infection was significantly associated with hypochlorhydria (OR 2.91, [1.02-7.75], p=0.046). While single infection with HIV was not significantly independently associated with hypochlorhydria. *H*. *pylori* and HIV co-infection was more strongly associated with hypochlorhydria (OR 6.25, [1.33-29.43], p=0.020) than either infection alone, suggesting an additive effect of co-infection. HIV infection was associated with higher serum gastrin levels (91.3pM vs. 53.1pM, p=0.040), while *H*. *pylori* infection was not (63.1pM vs. 55.1pM, p=0.610). Irrespective of *H*. *pylori* and HIV status, most patients (>90%) exhibited pangastritis. Only three patients had histological evidence of gastric atrophy, of which only one was HIV-infected.

**Conclusion:**

*H*. *pylori* infection was associated with fasting hypochlorhydria, while HIV was not independently associated. HIV and *H*. *pylori* co-infection, however, was more strongly associated with hypochlorhydria than *H*. *pylori* infection alone. The mechanism of this apparent additive effect between HIV and *H*. *pylori* remains unclear, but appears to be related to chronic pangastritis rather than gastric atrophy, and associated with hypergastrinaemia in HIV-infected individuals.

## Introduction

*Helicobacter pylori* is a gram-negative, spiral bacterium that colonises the gastric mucosa. It is typically acquired in childhood and persists throughout life in at least 50% of the world's population [1]. Since its discovery by Marshall and Warren in 1983, [2] overwhelming evidence has confirmed that *H*. *pylori* induces inflammation of the gastric mucosa through humoral and cellular immune mechanisms. Over time this leads to the development of chronic active gastritis, and in some cases peptic ulceration and gastric adenocarcinoma [1,3,4]. The host immune system is usually ineffective in eradicating the infection, yet the host inflammatory response causes a functional disruption to acid homeostasis, especially when the corpus mucosa is involved [2,5]. An important consequence of chronic *H*. *pylori* gastritis in some individuals is therefore low gastric acid output and hypochlorhydria [6]. Eradication of *H*. *pylori* may reverse this hypochlorhydria if atrophic gastritis has not already become established [1,7].

The association between *H*. *pylori* infection and hypochlorhydria is important because hypochlorhydria has been associated with increased susceptibility to enteric pathogens [2,8,9] and reduced drug absorption [1,3,4,10]. Hypochlorhydria has also been found to increase with age and to be commoner among males [2,5,11,12].

Human immunodeficiency virus (HIV) infection leads to a progressive loss of CD4+ T cells [6,13]. This is seen especially in the mucosa of the gastrointestinal tract, where most CD4+ T cells reside [14]. HIV infection is also associated with marked chronic inflammation of the gastric mucosa [15,16] and several studies have demonstrated that it is associated with low acid secretion and elevated fasting gastric pH [17–20]. One American study found that 93% (27/29) of HIV positive patients had a raised gastric pH, with a mean pH of 5.9 compared to 2.9 (p<0.05) in HIV negative subjects [18]. The mechanism for hypochlorhydria remains unknown, but a number of different hypotheses have been suggested. These include an autoimmune phenomenon (as high rates of anti-parietal antibodies are found in HIV patients) [18], that chronic infection and/or inflammation have a general inhibitory effect on acid secretion [21] or that the effect is due to an interaction with *H*. *pylori*.

At present the role of *H*. *pylori* infection in the gastrointestinal mucosa of HIV positive patients has not been well defined [22]. What is clear is that HIV infected subjects with gastric hypoacidity have significant drug malabsorption [23], and that this can sometimes be corrected by eradication of *H*. *pylori* [24].

One study found that over 90% of patients with AIDS also had histological chronic active gastritis. However this did not appear to be explained by dual infection, as *H*. *pylori* was present in only a minority of AIDS patients with chronic active gastritis in a Western setting. Chronic gastritis appeared as flattening of the surface epithelium and foveolar and shorter gastric pit length on histology. There was also a reduction in the number of lymphocytes and plasma cells in the lamina propria of HIV positive patients [16].

Gastrin is a peptide hormone that stimulates gastric acid (HCl) secretion by gastric parietal cells. Its release by G-cells in the gastric antrum is stimulated by the presence of food in the gastric lumen. Gastrin release is regulated by feedback inhibition via somatostatin from adjacent D-cells in the presence of a low gastric pH [25]. High fasting circulating concentrations of gastrin are often found in patients with chronic gastritis (usually secondary to *H*. *pylori* infection or pernicious anaemia) [26]. Chronic atrophic gastritis also in particular leads to the loss of acid secreting parietal cells, hypochlorhydria and hypergastrinaemia. *H*. *pylori* infection itself can also cause relatively mild hypergastrinaemia [27]. Several studies have reported higher serum gastrin concentrations among HIV positive patients [20,28] while one study reported that HIV status had no effect on serum gastrin levels [21].

We aimed to study the prevalence of hypochlorhydria (fasting pH>4) in symptomatic patients referred for gastroscopy in Malawi, and to investigate to what extent this was associated with *H*. *pylori* and/or HIV infections. In particular we aimed to identify whether HIV infection alone or as a co-infection with *H*. *pylori* was implicated in causing hypochlorhydria, and to understand the mechanism by studying histological changes and gastrin levels.

## Methods

We undertook a prospective descriptive study at the Queen Elizabeth Central Hospital, Blantyre, Malawi over 5 months. We recruited sequential symptomatic adults referred from the out-patient department for gastroscopy. We excluded patients under 18 years and those with evidence of an acute upper gastrointestinal bleed, a history of varices resulting from portal hypertension, or symptoms of dysphagia (given the high prevalence of oesophageal carcinoma in this setting). Patients with HIV who were established on antiretroviral therapy (ART) were recruited as an exploratory group, but were subsequently excluded from the main analysis per protocol. Ethical approval for the study was given by the University of Malawi College of Medicine Research and Ethics Committee (COMREC reference P.08/10/969), and all participants gave written informed consent. A medical history was taken, including basic sociodemographic details, smoking, alcohol and medication history and the ‘Shortened Leeds Dyspepsia Questionnaire’ was completed [29]. A 10ml fasting blood sample was taken for Rapid HIV testing (Uni-gold [Trinity Biotech Plc, Wicklow, Ireland] and Determine [Alere Ltd, Stockport, UK]. These two tests were required to be concordant to confirm HIV infection. Serum was stored at -80°C for later analysis of serum gastrin concentration.

All patients had a gastroscopy (after fasting for at least six hours), during which gastric fluid was aspirated using a 20ml syringe through the endoscope channel, after the channel had been emptied and dried by flushing with air. Fluid was tested using pH paper (Hydrion, Micro Essential Laboratory, USA). Gastric fluid pH >4 was defined as hypochlorhydria. Two biopsies were taken from the gastric antrum (within 1 inch of the pylorus) and two from the gastric body (greater curve, proximal third), for histology. All endoscopists had been trained and monitored using reproducible direct observation endoscopy skills training tools, as part of an international training programme, and were trained in upper gastrointestinal lesion recognition [30].

Histological analysis was performed by a consultant gastrointestinal histopathologist (TA) who determined the *H*. *pylori* infection status and extent of any inflammatory and atrophic gastritis, using the modified Sydney classification [31]. Gastrin analysis was performed by radioimmunoassay using antibody L2, which reacts with G17 and G34, but not progastrin or gly-gastrins as previously described [32].

SPSS v19 (SPSS Inc, Chicago, IL) was used for statistical analyses. Summary statistics were calculated and then contingency tables were used to investigate univariate associations, significance being assessed using the standard chi-squared test. Logistic regression was then used to investigate individual and combinatorial factors, using a stepwise approach. Statistical significance was defined at 5% (p = 0.05).

## Results

### Patient characteristics and clinical history

104 patients were initially recruited but nine were subsequently excluded from further analysis, per protocol, because they were HIV positive and already established on ARV medication. This was because we primarily aimed to study the effect of *H*. *pylori* and untreated HIV infection on fasting gastric acidity. The 9 patients on ARV medication were analysed separately, as an exploratory group.

The remaining 95 patients had a mean age of 33 years (sd 13.2), ranging from 18–78 years, and 55 (57.9%) patients were female. There were 6 (6.3%) current smokers, of whom 4 were male, and 14 (14.7%) patients regularly consumed alcohol of whom 9 were male. Overall, 68 (71.6%) patients were *H*. *pylori* positive and 17 (17.9%) were HIV positive and had not been established on antiretroviral medication (ARV). All HIV rapid tests were concordant by both methods.

Most patients were referred to gastroscopy after empirical outpatient treatment with combinations of anti-secretory medication and antibiotics for dyspeptic or reflux symptoms, in an attempt to eradicate presumed *H*. *pylori* infection.

Prior antibiotic usage was the norm, with 89 (93.7%) patients having taken at least one course within the last year. The majority of patients received amoxicillin with a minority having taken additional courses of metronidazole or ciprofloxacin. None of the prescriptions represented an accepted *H*. *pylori* eradication regimen [33]. Due to the time between the outpatient appointment and gastroscopy, only 3 (3.2%) patients had taken antibiotics during the two weeks prior to enrolment.

Anti-secretory medications had also commonly been used in the year prior to gastroscopy. 93 (97.8%) patients had received some form of treatment; omeprazole (n = 69), magnesium trisilicate (n = 22) or cimetidine (n = 2). As most prescriptions were only for a few weeks, only 5 (5.2%) patients had taken such treatment in the two weeks prior to gastroscopy.

### The indication for gastroscopy

The commonest indication for gastroscopy was epigastric pain, which accounted for 87 (91.2%) referrals. Two patients were investigated for anaemia, two for previous haematemesis, two for odynophagia, one for reflux symptoms and one to confirm peptic ulcer healing.

Patients’ symptoms were interrogated using the ‘Leeds Dyspepsia Questionnaire’. The commonest symptoms were reflux related (heartburn/regurgitation), which accounted for 46 (48.4%) patients. Of the remaining patients, ulcer-like (indigestion) symptoms were described by 31 (32.6%), dysmotility-like (nausea) symptoms by 4 (4.2%) and 14 (14.7%) patients could not identify a predominant symptom.

### Gastroscopy findings

The commonest endoscopic findings in the stomach were erythematous (33.7%) and atrophic (31.6%) gastritis. Normal gastric mucosa was seen in 33.7% of patients. Significant pathological findings included two cases of oesophageal varices, one gastric ulcer and one gastric cancer, Table 1. Endoscopic appearances of gastritis were seen in 63.2% (43/68) of *H*. *pylori* infected patients and 64.7% (11/17) of HIV infected patients.

**Table 1:** Findings at gastroscopy; the examination was entirely normal in 27 (26%) patients.

Gastroscopy findings	No. (%)
*Oesophagus*	
Normal	78 (81.7)
Candida	6 (6.7)
Oesophagitis	6 (6.7)
Hiatus hernia	3 (2.9)
Varices	2 (1.9)
*Stomach*	
Normal	31 (32.6)
Erythematous gastritis	32 (33.7)
Atrophic gastritis	30 (31.6)
Gastric ulcer	1 (1.1)
Gastric cancer	1 (1.1)
*Duodenum*	
Normal	84 (88.4)
Duodenitis	6 (6.3)
Not entered	5 (5.3)

### Patient characteristics and fasting gastric pH

In the HIV positive group, 76.5% (13/17) were also *H*. *pylori* positive (Table 2), which was not significantly higher than 70.5% (55/78) in the HIV negative group, p = 0.622. The mean gastric fluid pH overall was 4.7 (sd 2.4) and 55.8% (53/95) of patients were hypochlorhydric (defined as a fasting gastric pH>4). Table 3 shows the mean pH readings by age (< or > = 40 years), gender and *H*. *pylori* and HIV infection status.

**Table 2:** The number of patients who were infected with HIV or *H*. *pylori*.

	HIV positive	HIV negative	Total
*H*. *pylori* infected	13	55	68
*H*. *pylori* uninfected	4	23	27
Total	17	78	95

**Table 3:** Mean gastric pH values by age, gender, *H*. *pylori* and HIV status.

Variable	pH, mean (sd)	p value
**Age group** (years)		
<40 (n = 77)	4.7 (2.4)	0.769
40 and over (n = 18)	4.9 (2.5)	
**Gender**		
Male (n = 40)	4.3 (2.5)	0.107
Female (n = 55)	5.1 (2.3)	
***H*. *pylori***		
Negative (n = 27)	4.0 (2.2)	0.061
Positive (n = 68)	5.0 (2.5)	
**HIV**		
Negative (n = 78)	4.6 (2.4)	0.117
Positive (n = 17)	5.6 (2.5)	

Following univariate and multivariate analysis (Table 4), *H*. *pylori* infection was the only variable that was significantly and independently associated with hypochlorhydria (OR 3.2 (1.1–9) p = 0.029), with 63.2% of *H*. *pylori* positive individuals being hypochlorhydric, compared to 37.0% of *H*. *pylori* negative individuals. There was also a non-significant trend (p = 0.061) towards higher pH readings in *H*. *pylori* infected patients (Table 3). HIV infection was not independently associated with hypochlorhydria (Table 4) or with high gastric fluid pH (Table 3).

**Table 4:** Univariate and binary logistic regression by factors potentially associated with hypochlorhydria.

Variable	Hypochlorhydria/Total (%)	Unadjusted (univariate)			Adjusted (multivariate)		
		OR	CI	p value	OR	CI	p value
**Age group** (years)							
<40	42/77 (54.5)	1	-	-	1	-	-
40 and over	11/18 (61.1)	1.31	0.46–3.73	0.614	1.01	0.32–3.16	0.989
**Gender**							
Male	18/39 (46.2)	1	-	-	1	-	-
Female	35/56 (62.5)	1.94	0.85–4.46	0.116	1.72	0.69–4.26	0.242
***H*. *pylori***							
Negative	10/27 (37.0)	1	-	-	1	-	-
Positive	43/68 (63.2)	2.92	1.16–7.36	0.023	3.19	1.13–9.03	0.029
**HIV**							
Negative	41/78 (52.6)	1	-	-	1	-	-
Positive	12/17 (70.6)	2.08	0.67–6.54	0.208	1.89	0.56–6.32	0.304

### Effect of single infection or co-infection with H. pylori and HIV on hypochlorhydria

There were 13 patients who had dual *H*. *pylori* and HIV infection and this group had a higher proportion of hypochlorhydric patients (76.9%) than the groups with a single infection (*H*. *pylori* 60.0%, HIV 50.0%) or no infection (34.8%) (Table 5).

**Table 5:** Age, gender and hypochlorhydria in subjects singly infected or co-infected with *H*. *pylori* or HIV.

HP, HIV combination	Age group (years), n (%)		Gender, n (%)		Hypochlorhydria, n (%)	
	<40	40 and over	Male	Female	Yes	No
HP–ve & HIV–ve	20 (87.0)	3 (13.0)	15 (56.5)	10 (43.5)	8 (34.8)	15 (65.2)
HP+ve & HIV–ve	41 (74.5)	14 (25.5)	30 (54.5)	25 (45.5)	33 (60.0)	22 (40.0)
HP–ve & HIV+ve	4 (100)	0	4 (100)	0	2 (50.0)	2 (50.0)
HP+ve & HIV+ve	12 (92.3)	1 (7.7)	9 (69.2)	4 (30.8)	10 (76.9)	3 (23.1)

**Fig 1:**
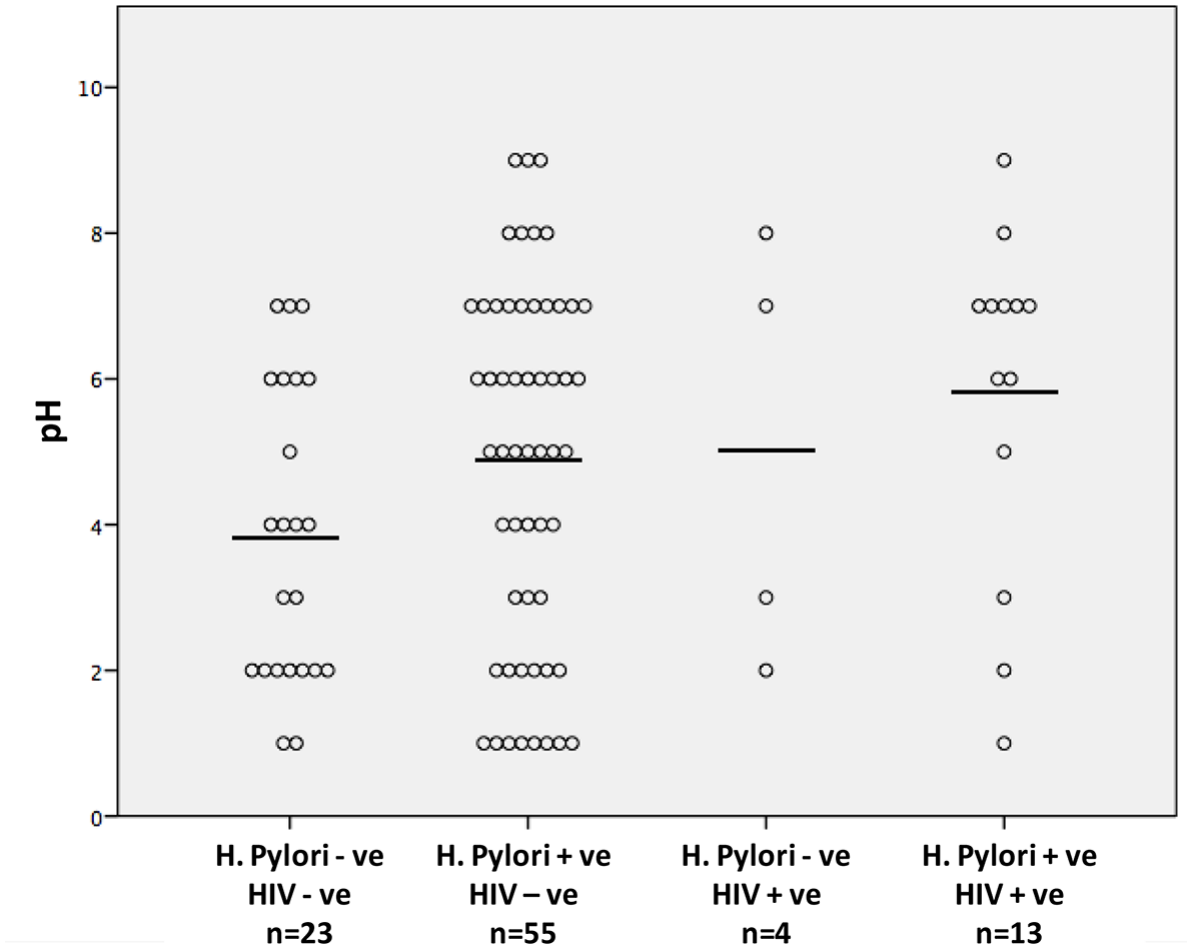
The scatterplot displays the absolute pH values for the four groups in relation to *H*.*pylori* and HIV infection status.

In a binary logistic regression analysis, infection with *H*. *pylori* alone was associated with a significantly increased OR for hypochlorhydria (OR 2.81 (1.02–7.75), p = 0.046) compared to uninfected individuals, (Table 5). However, the highest and most significant risk for hypochlorhydria was seen in patients who were co-infected with *H*. *pylori* and HIV, OR = 6.25 (1.33–29.43) (p = 0.020) (Table 6). The mean gastric pH for uninfected patients was 3.8 (CI: 2.0), for patients only infected with *H*. *pylori* it was 4.9 (CI: 2.5) and for patients only infected with HIV it was 5.0 (CI: 2.9). For patients co-infected with HIV and *H pylori* it was 5.8 (CI: 2.4) (Fig 1), but there were no statistically significant differences between these groups.

**Table 6:** Binary logistic regression analysis investigating the impact of single infection or co-infection with *H*. *pylori* (HP) and HIV on the odds of having hypochlorhydria.

Variable	Hypochlorhydria/Total (%)	Unadjusted (univariate)		
		OR	CI	p value
**HP/HIV status**				
HP–ve & HIV–ve	8/23 (34.8)	1	-	-
HP+ve & HIV–ve	33 /55 (60.0)	2.81	1.02–7.75	0.046
HP–ve & HIV+ve	2 /4 (50.0)	1.89	0.22–15.93	0.565
HP+ve & HIV+ve	10 /13 (76.9)	6.25	1.33–29.43	0.020

In order to understand the possible mechanisms by which HIV and *H*. *pylori* might act together in an additive manner to cause hypochlorhydria, we evaluated gastric histology and compared fasting serum gastrin concentrations.

### Gastric Histopathology

Biopsies from the gastric antrum and body allowed gastritis to be described as antral predominant (Type B), corpus predominant (Type A) or pangastritis (Type AB). The degree of inflammation, whether minimal (superficial) or full thickness and the type, whether chronic (mononuclear infiltrate) or chronic active (mononuclear infiltrate with neutrophils) was also recorded (Table 7). All biopsies were also assessed for evidence of gastric atrophy, intestinal metaplasia and the presence of *H*. *pylori* organisms.

**Table 7:** Histological assessment of antral and corpus (body) biopsies from all subjects.

Antral					Body			
	Normal	Minimal Chronic Inflammation	Chronic Inflammation	Chronic Active Inflammation	Normal	Minimal Chronic Inflammation	Chronic Inflammation	Chronic Active Inflammation
HP-ve, HIV-ve (n = 23)	4 (17.4)	10 (43.6)	6 (26.1)	3 (13.0)	1 (4.3)	11 (47.8)	10 (43.6)	1 (4.3)
HP+ve, HIV-ve (n = 55)	1 (1.8)	6 (10.9)	20 (36.4)	28 (50.9)	0	10 (18.2)	26 (47.3)	19 (34.5)
HP-ve, HIV +ve (n = 4)	0	0	4 (100)	0	0	0	4 (100)	0
HP+ve, HIV+ve (n = 13)	1 (7.7)	1 (7.7)	4 (30.8)	7 (53.8)	0	1 (7.7)	4 (30.8)	8 (61.5)

Pangastritis was a near-universal finding in our patient cohort. Overall 99.0% (n = 94) of gastric body biopsies and 93.7% (n = 89) of antral biopsies showed some evidence of chronic inflammation. Over 90% of *H*. *pylori* or HIV infected patients had evidence of chronic inflammation, whether minimal or full thickness. Even in the *H*. *pylori*-negative and HIV negative group, 82.6% (19/23) of patients showed some evidence of chronic stomach inflammation.

We found that chronic active pangastritis was absent from all mono HIV infected patients (0/4), whereas over half of mono *H*. *pylori* infected patients had active inflammation. Secondly, in *H*. *pylori* patients, active inflammation was more commonly seen in the antrum rather than body (51% vs. 35%). Patients with dual *H*. *pylori* and HIV infection also showed a mixture of chronic and chronic active inflammation in both the antrum and body. Interestingly, none of the 4 patients infected only with HIV had evidence of active inflammation at either the antral or body sites (Table 7).

There were three cases of intestinal metaplasia and a further three cases of gastric atrophy. In only one of the cases of gastric atrophy was the patient HIV positive. The mean age of the three patients who had gastric atrophy was 52 years, which was older than the mean study age (34 years). Gastric atrophy appeared to be associated with *H*. *pylori* infection, which was found in all three patients, whose endoscopic findings showed one gastric ulcer, one case of erythematous gastritis and one normal study.

### Gastric pH and fasting serum gastrin concentrations

**Fig 2:**
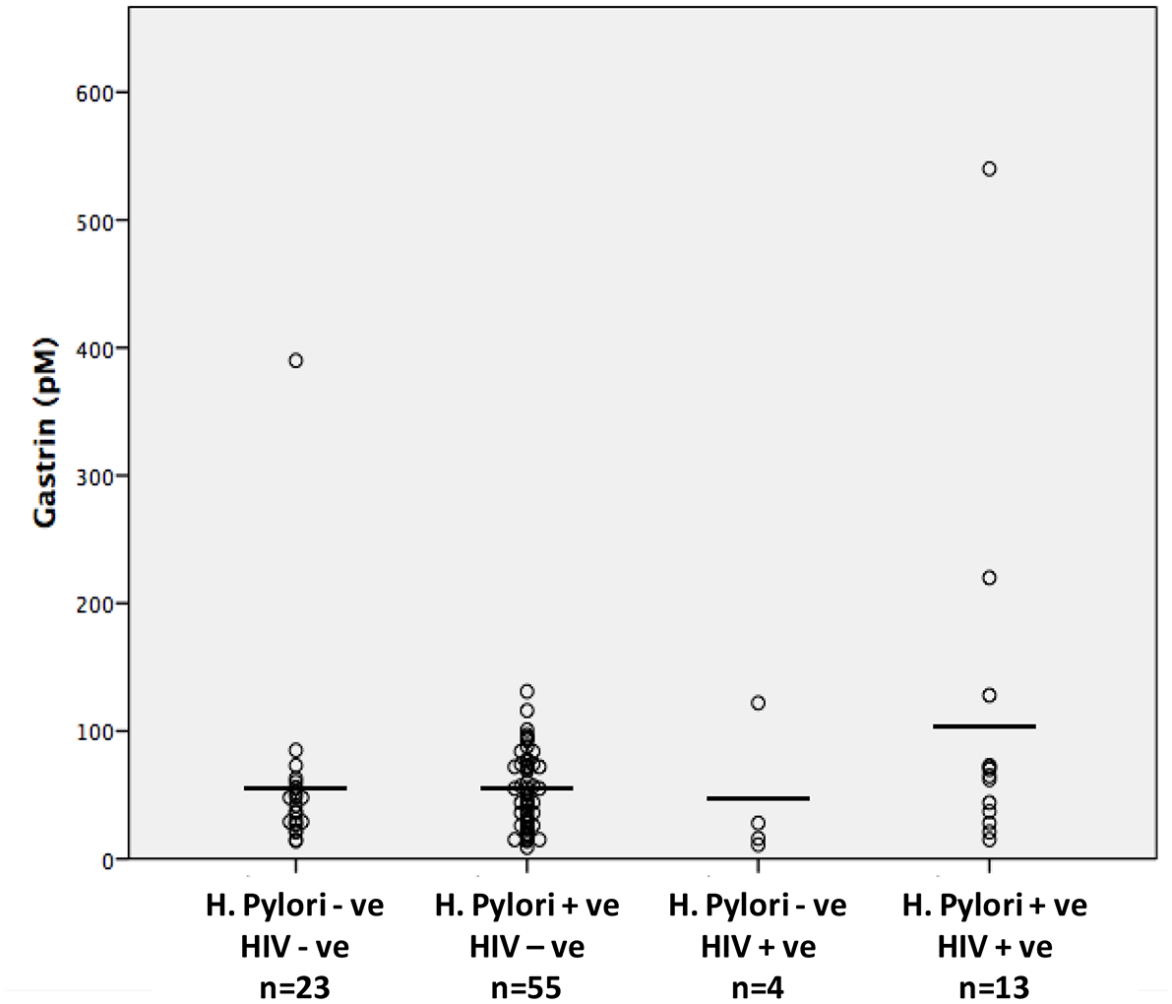
The scatterplot displays the serum gastrin (pM) levels for the four groups in relation to *H*.*pylori* and HIV infection status.

The mean fasting serum gastrin concentration was 61pM (sd 69.1) with a range between 9–540pM. As anticipated, patients with hypochlorhydria had significantly higher serum gastrin concentrations (77 vs. 41, p = 0.028). The mean fasting serum gastrin concentration for patients with *H*. *pylori* infection (63.1pM) was not significantly different from that in *H*. *pylori* negative patients (55.1pM, p = 0.610). However in patients with HIV infection, serum gastrin concentrations were significantly higher than in HIV-uninfected individuals (91.3 vs. 53.1, p = 0.040). Mean serum gastrin concentration in co-infected individuals was even higher at 105pM (sd 141). While the mean gastrin level was highest in this co-infected group (H.pylori +ve HIV +ve), there were no significant differences seen between the uninfected, singly-infected and co-infected groups (Fig 2).

### Exploratory description of HIV-infected patients on ART

Of the nine HIV positive patients on ARVs, 5 were *H*. *pylori* co-infected. These 9 had a mean gastric fluid pH of 3.1 and fasting serum gastrin concentration of 55pM, values that were almost identical to HIV negative patients.

## Discussion

We have shown that in a population of dyspeptic Malawian adults, *H*. *pylori* infection was a significant risk factor for hypochlorhydria. Furthermore, while infection with HIV alone was not associated with hypochlorhydria, it appeared to have an additive effect with *H*. *pylori* infection, to increase the likelihood that a patient would be hypochlorhydric. These findings are important because hypochlorhydria is associated with an increased risk of enteric infection and impaired drug absorption, both of which are important factors in maintaining the health of HIV infected patients [34]. Our findings offer an insight into a poorly understood and potentially reversible problem.

Chronic infection with *H*. *pylori* is well known to cause hypochlorhydria, through inflammatory interruption of acid production. HIV infection has also previously been shown to cause hypochlorhydria and elevated serum gastrin concentrations, but the findings of studies have been inconsistent, and the mechanism responsible for these effects is unknown [35]. We found no statistically significant independent association of HIV, per se, with either pH or hypochlorhydria. We did however observe an additive effect in patients coinfected with HIV and *H*. *pylori* causing increased hypochlorhydria, and significantly increased fasting serum gastrin concentrations in HIV positive patients.

While most HIV infected patients were also infected with *H*. *pylori* (76.9%), the prevalence of *H*. *pylori* infection was not significantly higher than in the HIV negative group (70.5%). This is in line with other studies [36–38], some of which have actually found lower rates of *H*. *pylori* infection in patients with progressive HIV infection and low CD4 counts [39,40].

Previous treatment for *H*. *pylori* infection is a potential confounder in this study. We found that, because of constrained resources in Malawi, prior attempts at eradication therapy for *H*. *pylori* were largely empirical, and never involved a recognized or effective regime, in keeping with our previous findings [41]. Only 5% of patients were on antibiotics or acid suppression during the fortnight prior to the study, and previous or current eradication therapy is therefore unlikely to have directly affected our findings.

In order to understand potential mechanisms for this synergy, we evaluated histological samples for *H*. *pylori* and HIV positive patients. Histological analysis of biopsies from both the antrum and body demonstrated that chronic pangastritis (Type AB) was the norm in this patient cohort, affecting 90% of patients irrespective of infection status. Given the ubiquitous finding of chronic inflammation across all groups, it was not possible to demonstrate a clear association between histological findings and other patient variables. However chronic *active* inflammation, indicating the presence of neutrophils, did allow us to describe apparent differences between groups by infection profile. Chronic active pangastritis is a classical finding in *H*. *pylori* infection; we found it was not present in mono HIV infected patients but was seen at both sites in those who were mono infected with *H*. *pylori*, and was also seen at both sites in co-infected. Interestingly, a Brazilian study found that antral chronic active gastritis was associated with both HIV and *H*. *pylori* infection, but that in the body of the stomach, chronic active gastritis was seen in HIV-infected patients, independently of *H*. *pylori* infection [40].

We also described differences in the distribution of inflammation in the stomach. *H*. *pylori* infection alone was more likely to lead to antral gastritis (which might not be expected to cause a significant increase in serum gastrin concentrations), while pangastritis (more likely to cause increased serum gastrin concentrations) was associated with co-infection with HIV.

We were able to establish that gastric atrophy was not the cause of hypochlorhydria in the majority of our patients. There were only three case of gastric atrophy in our cohort and only one of these cases was also HIV positive. This finding is consistent with the study of Kelly et al. that was performed in a similar geographical setting [35], although their study did not report on *H*. *pylori* infection. This is important because when atrophy has not become established, then *H*. *pylori* eradication may reverse the hypochlorhydria.

Our study’s finding of a high prevalence of chronic inflammation, but a low prevalence of gastric atrophy has also been shown in other studies from African populations [42]. Therefore, the most likely reason for the rise in gastric pH with dual *H*. *pylori* and HIV infection is that HIV infection results in greater chronic pangastric inflammation and resultant reduced gastric acid secretion, as evidenced by the significantly higher serum concentrations of gastrin in HIV infected patients.

Hypochlorhydria increases the risk of acquiring enteric infections and reduces drug absorption (for example anti-retroviral therapy and antibiotics). Our small, exploratory group of individuals established on ART showed a lower gastric pH, suggesting that ART treatment might reverse the apparent synergistic effect of HIV and ameliorate hypochlorhydria. We can also speculate that eradication of *H*. *pylori* would reduce hypochlorhydria, even in the presence of HIV. Either of these strategies might reduce the incidence of enteric infections and improve absorption of medications. Longer-term follow up cohort studies would be required to evaluate these possibilities.

Interestingly one small study has shown that eradication of *H*. *pylori* also facilitates immune reconstitution in HIV-1-infected immunological non-responders [43]. Similarly, Shelton et al showed in a small study that eradication of *H*. *pylori* in hypochlorhydric HIV patients increased delavirdine absorption [24]. However eradication regimes would need to be more effective in our setting, as around 90% of our patients had already received empirical attempts at *H*. *pylori* eradication, using unproven eradication regimes, prior to their gastroscopy, yet *H*. *pylori* infection rates were still very high at 70% [41].

Weaknesses of this study include the inclusion of only symptomatic (mainly dyspeptic) patients and the lack of assessment of HIV disease stage. Due to the relatively small size of the study, there were power limitations. A larger study may have been able, for example, to detect a clinically significant difference in the prevalence of *H*. *pylori* infection between HIV positive and HIV negative individuals. The small number of co-infections also limited our ability to explore the relationship statistically, and larger study might be able to add statistical resolution to these findings. Most patients had been prescribed apparently ineffective attempts at *H*. *pylori* eradication, but it remains possible that some of the subjects who were classified as *H*. *pylori* negative in this study may have previously been infected but successfully treated. It could be argued, that we should have excluded the three patients who received antibiotics and five patients who took anti-secretory medications in the two weeks before the gastroscopy. In addition, assessing pH using a single fasting gastric fluid sample is not as sensitive in detecting hypochlorhydria by measuring acid secretion. However the latter was not practicable in the Malawian setting.

In conclusion, mono infection with *H*. *pylori* but not HIV was associated with hypochlorhydria. This risk for hypochlorhydria was strongest, however, in patients with dual *H*. *pylori* and HIV infection. Co-infected patients also demonstrated the highest mean gastric fluid pH, suggesting an additive effect between the two infections to increase hypochlorhydria. The mechanism of HIV-related induced hypochlorhydria in this setting remains unclear, but appears to be associated with a chronic pangastritis as evidenced by the histology, and resulting high fasting serum gastrin concentrations, and does not appear to be caused by gastric atrophy. This suggests that the finding is reversible, and merits larger studies to confirm this finding, and further studies of its impact and treatment.

## Supporting Information

S1 file Supplementary data file containing the data collected in the preparation of this manuscript. (SAV)
